# Supplementary material for: Porcine nasal organoids to model interactions between the swine nasal microbiota and the host
Source: Microbiome. 2025 May 22;13:131. doi: 10.1186/s40168-025-02088-9 (PMC12100890; doi:10.1186/s40168-025-02088-9)
Supplement: Supplementary file 2 — Supplementary Material 1. [file 40168_2025_2088_MOESM1_ESM.docx]

Supplementary Materials for

**Porcine nasal organoids to model interactions between the swine nasal microbiota and the host**

Laura Bonillo-Lopez^#^, Noelia Carmona-Vicente^#^*, Ferran Tarrés-Freixas,

Karl Kochanowski, Jorge Martínez, Mònica Perez, Marina Sibila,

Florencia Correa-Fiz, Virginia Aragon

# These authors contributed equally to this work

*Corresponding author. Email: [noelia.carmona@irta.cat](mailto:noelia.carmona@irta.cat)

**This PDF file includes:**

Figures S1 to S11

**Other Supplementary Materials for this manuscript include the following:**

Movie S1


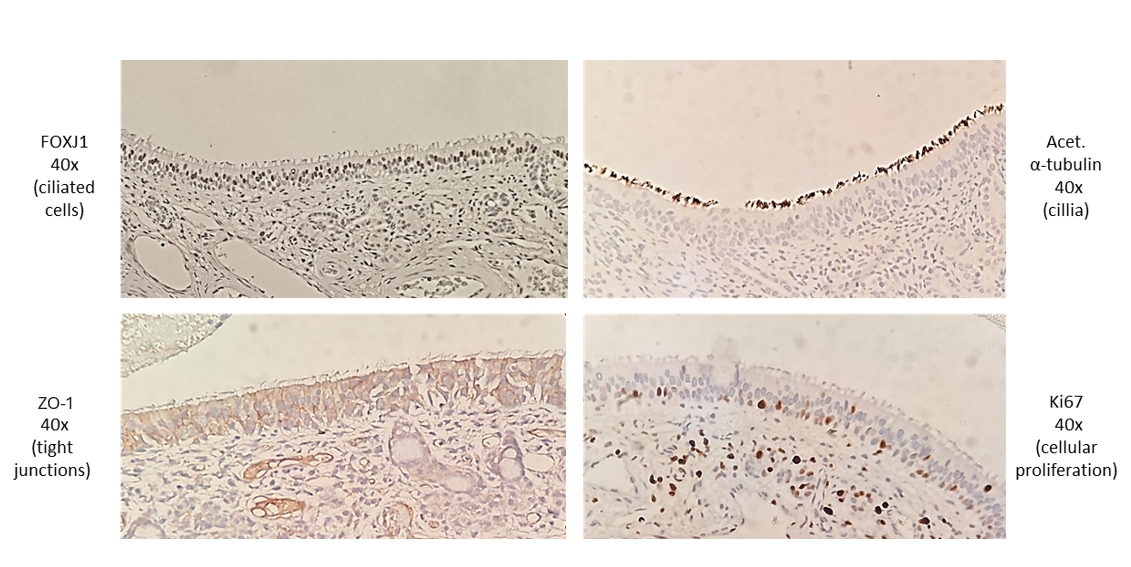


Figure S1. Characterization and cell marker expression in paraffin-embedded nasal turbinate from pigs by IHQ. Nasal turbinate tissue was used for immunohistochemistry staining. Antibodies against FOXJ1 and acetylated α-tubulin (ciliated cells, left and right upper panel, respectively), ZO-1 (tight junctions, left lower panel), and Ki67 (proliferative cells) were used to visualize the different cells markers expressed on the nasal airway epithelium from pigs. Images are representative of at least five similar images.


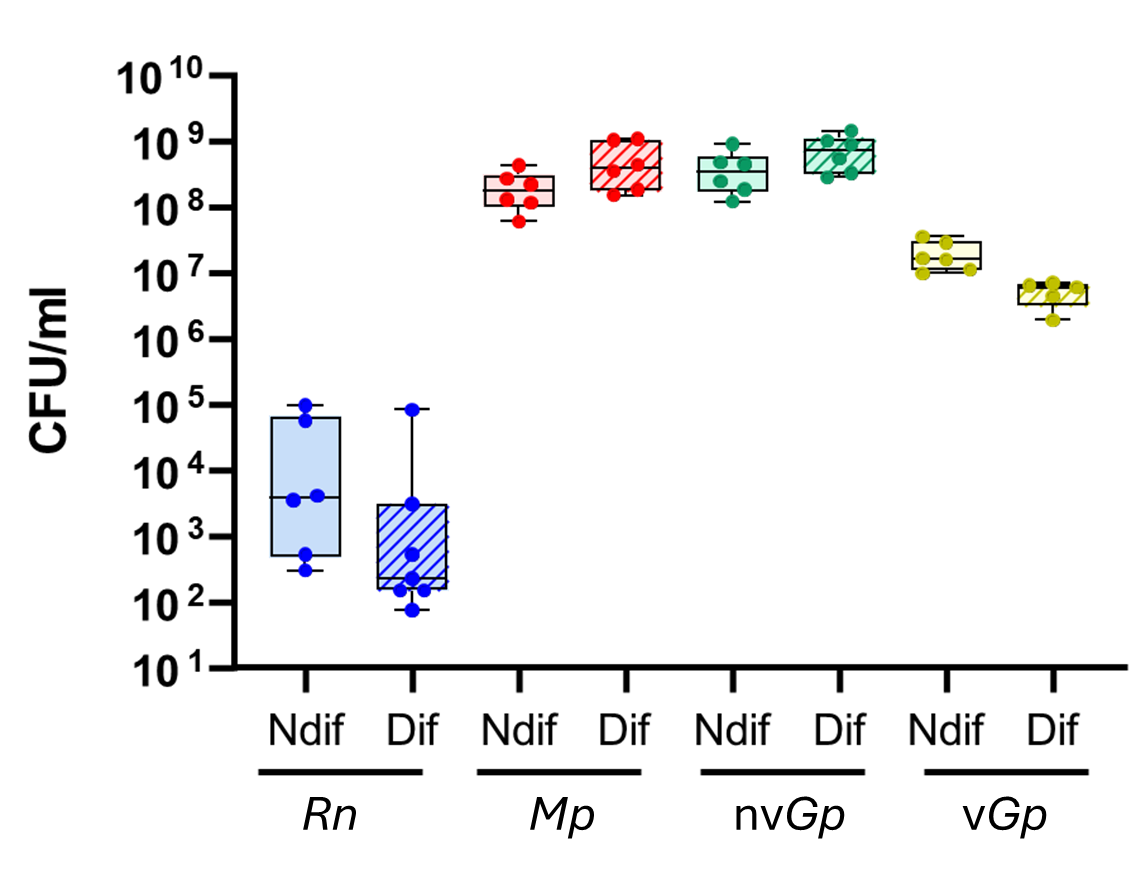


Figure S2. Bacteria found in the supernatant of non-differentiated and differentiated Porcine Nasal Organoids (PNOs) after overnight incubation. Non-differentiated (Ndif, plain pattern) and differentiated (Dif, stripped pattern) PNOs were overnight incubated with approximately 10^4^-10^5^ CFU/ml of *R. nasimurium* (*Rn*, in blue), 10^3^ CFU/ml of *M. pluranimalium* (*Mp*, in red), 10^3^ CFU/ml of non-virulent *G. parasuis* (nv*Gp*, in green) or 10^5^-10^6^ CFU/ml of virulent *G. parasuis* (v*Gp*, in yellow). After incubation, bacteria present in the supernatant were quantified by dilutions and plating. Each dot represents a replicate from three different experiments of PNOs coming from two animals. Two replicates were included in each experiment.


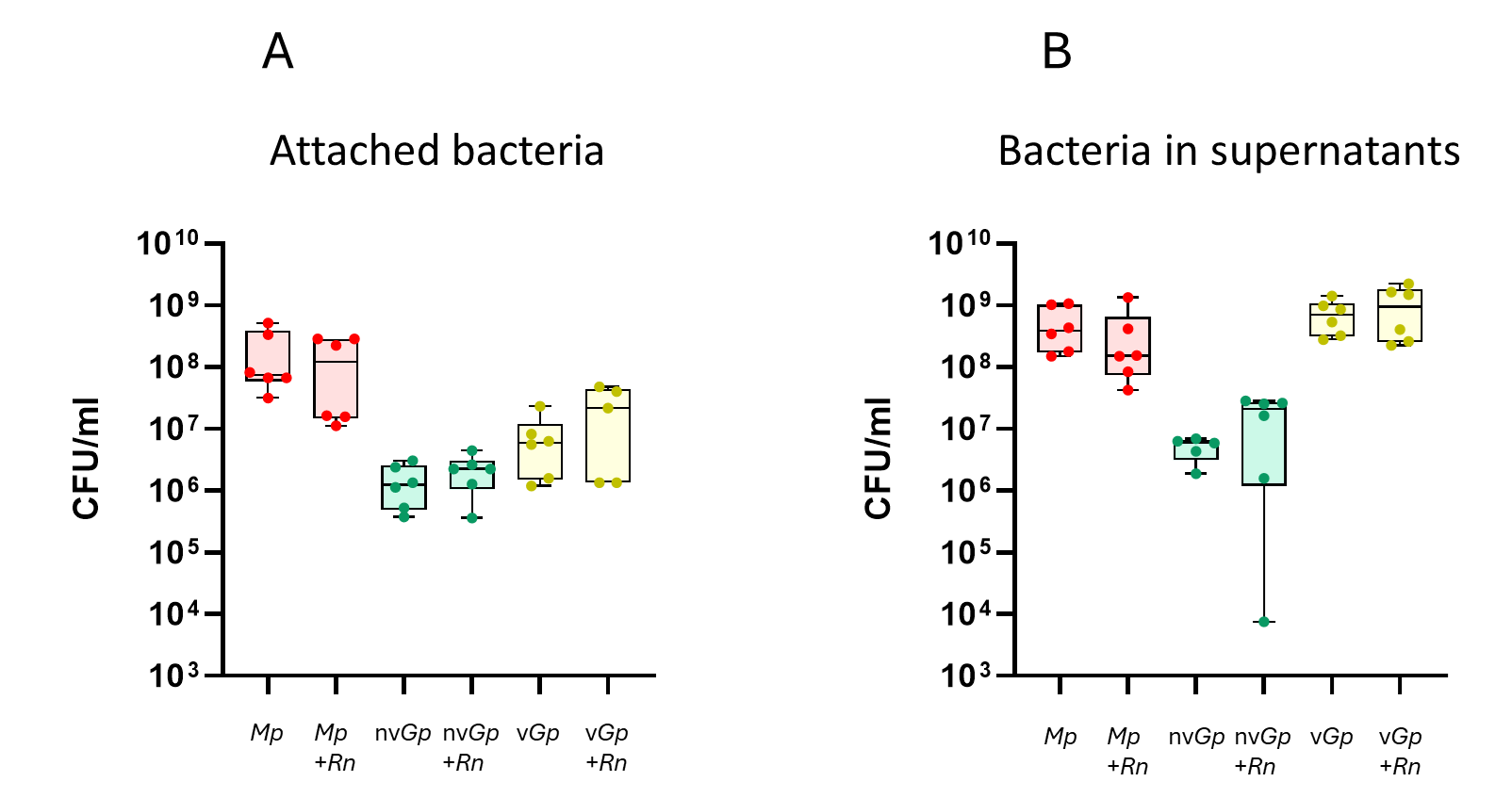


Figure S3. Co-culture with Rothia nasimurium in the Porcine Nasal Organoids (PNOs) after overnight incubation. Differentiated PNOs were incubated overnight with approximately 10^3^ CFU/ml of *Moraxella pluranimalium* (*Mp*), 10^3^ CFU/ml of non-virulent *Glaesserella parasuis* (nv*Gp*) or with 10^6^ CFU/ml of virulent *G. parasuis* (v*Gp*) individually or in combination with 10^5^ CFU/ml of *R. nasimurium* (*Rn*). a) As a measure of colonization capacity, attached *Mp* (in red), nv*Gp* (in green) and v*Gp* (in yellow) were quantified after individual incubation or co-cultured with *Rn*. b) Quantification of *Mp* (red), nv*Gp* (green) and v*Gp* (yellow) in the supernatant. Each dot represents a replicate from three different experiments of PNOs coming from two animals. Two replicates were included in each experiment.


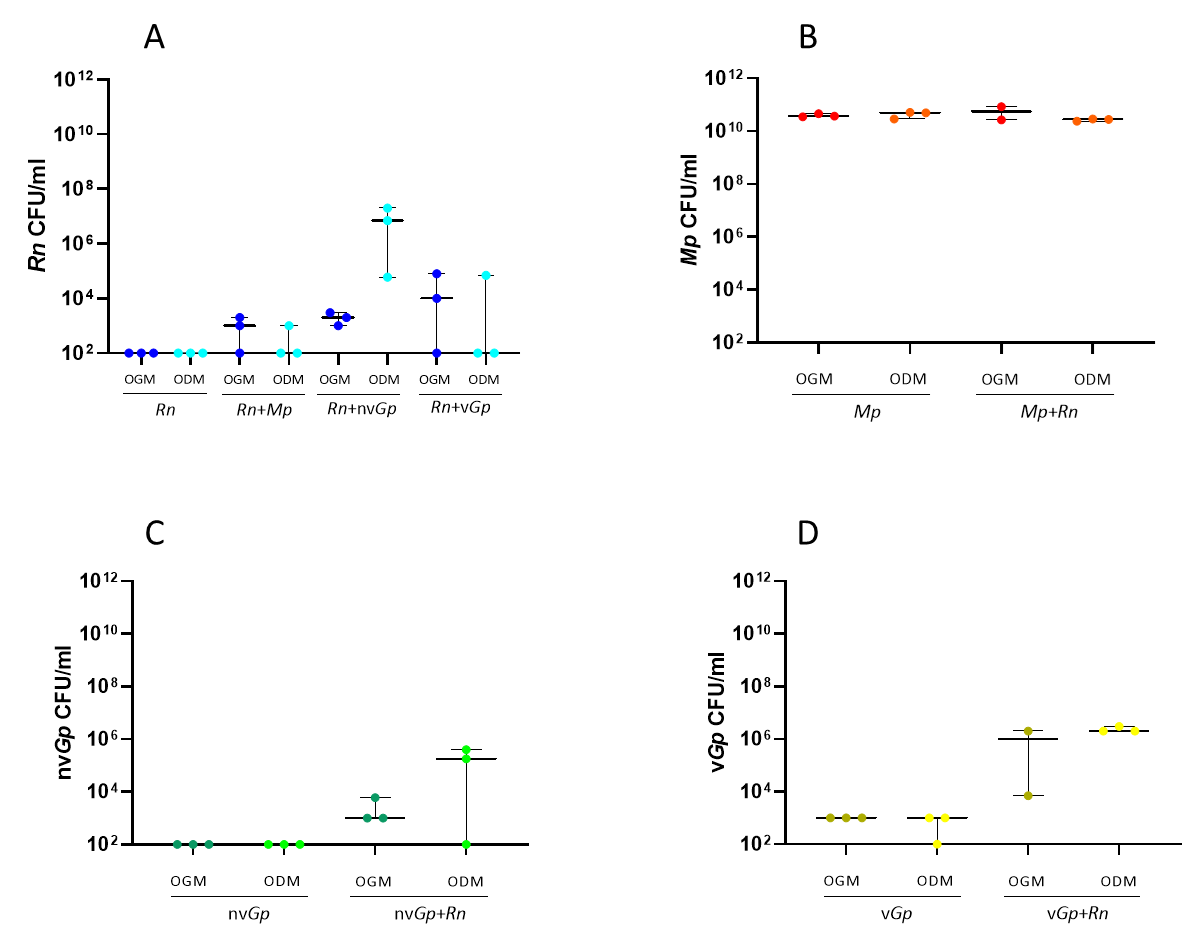


Figure S4. Overnight co-culture of bacterial strains in fresh organoid media. Bacterial growth (CFU/ml) after overnight incubation in fresh Organoid Growth Media (OGM) and fresh Organoid Differentiation Media (ODM). a) *Rothia nasimurium* (*Rn*) individually grown as well as in co-culture with *Moraxella pluranimalium* (*Mp*, *Rn*+*Mp*), non-virulent *Glaesserella parasuis* strain (nv*Gp*, *Rn*+nv*Gp*) and virulent *G. parasuis* strain (vGp, *Rn*+v*Gp*) in OGM (dark blue) and ODM (turquoise). b) *Mp* individually grown and in co-culture with *Rn* (*Mp*+*Rn*) in OGM (red) and ODM (orange). c) nv*Gp* individually grown and in co-culture with *Rn* (nv*Gp*+*Rn*) in OGM (dark green) and ODM (light green). d) v*Gp* individually grown and in co-culture with *Rn* (v*Gp*+*Rn*) in OGM (dark yellow), ODM (light yellow).


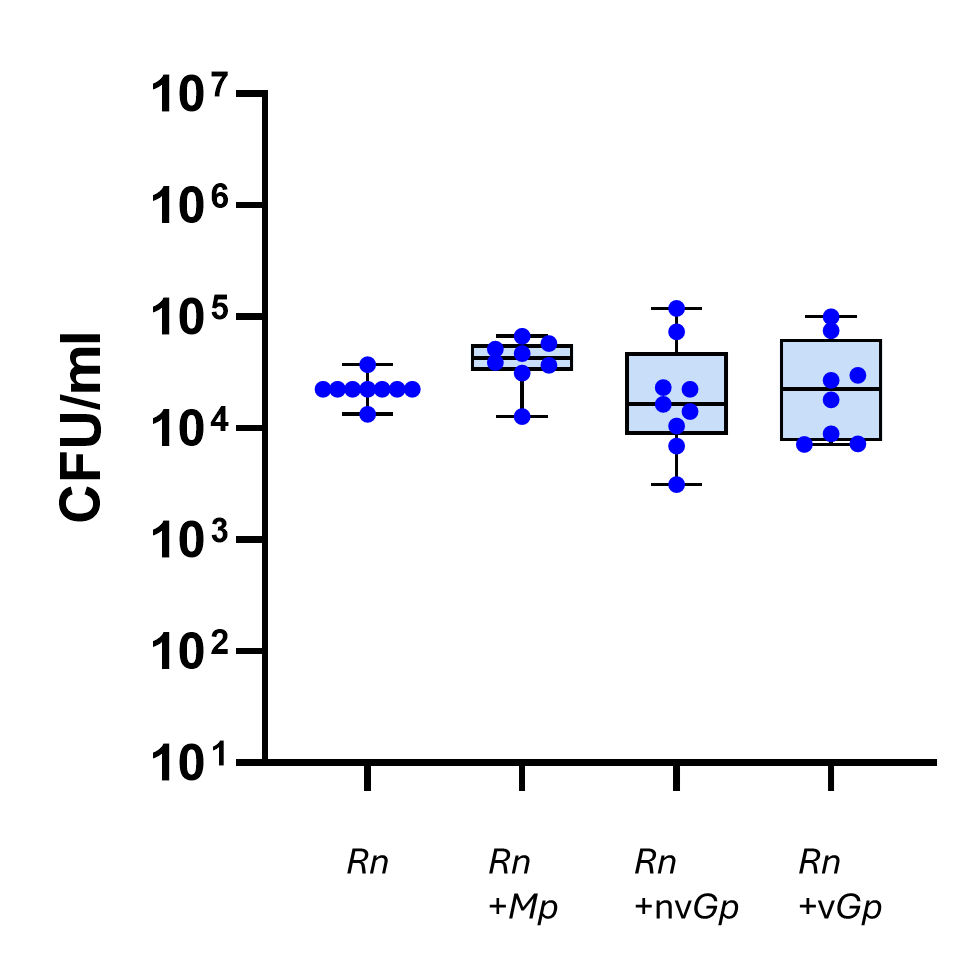


Figure S5. *Rothia nasimurium* adhesion after 2 h of incubation with differentiated porcine nasal organoids (PNOs) individually or in co-culture with selected strains. Differentiated PNOs were inoculated with 10^5^ CFU/ml of *R. nasimurium* (*Rn*) alone or in combination with approximately 10^3^ CFU/ml of *Moraxella pluranimalium* (*Mp*), 10^3^ CFU/ml of non-virulent *Glaesserella parasuis* (nv*Gp*) or with 10^6^ CFU/ml of virulent *G. parasuis* (v*Gp*). After 2 h of incubation, attached *Rn* was quantified by dilutions and plating, after washing to eliminate unbound bacteria. Three independent experiments were performed with duplicate wells and each dot in the plot represents individual well results.


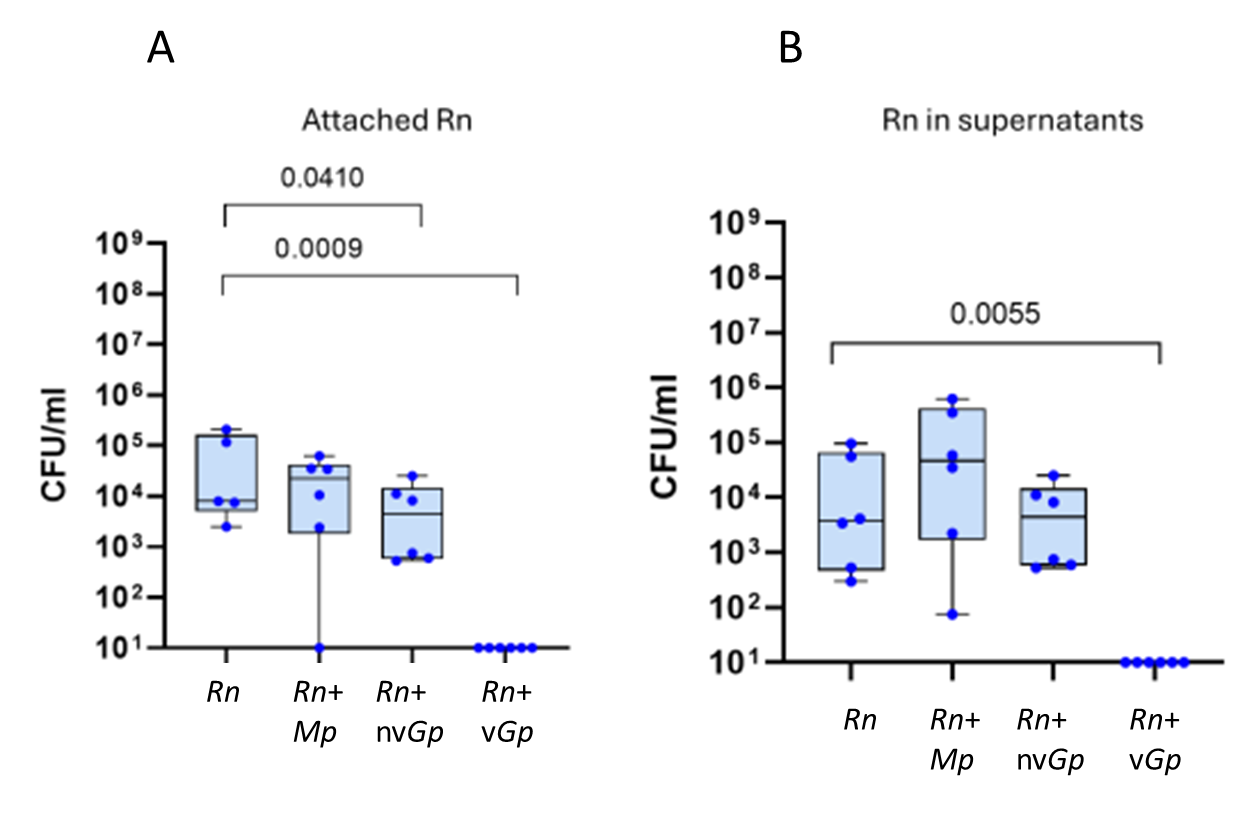


Figure S6. *Rothia nasimurium* load after overnight incubation with non- differentiated porcine nasal organoids (PNOs) individually or in co-culture with selected strains. Non-differentiated PNOs were inoculated with 10^4^ CFU/ml of *R. nasimurium* (*Rn*) alone or in combination with approximately 10^3^ CFU/ml of *Moraxella pluranimalium* (*Mp*), 10^3^ CFU/ml of non-virulent *Glaesserella parasuis* (nv*Gp*) or with 10^5^ CFU/ml of virulent *G. parasuis* (v*Gp*). After overnight incubation, attached *Rn* (A) and *Rn* in the supernatant (B) were quantified by dilutions and plating. Three independent experiments were performed with duplicate wells and each dot in the plot represents individual well results. Kruskal-Wallis multiple comparison with Benjamini, Krieger and Yekutieli post-hoc test was used to compare the bacterial concentrations. Significant differences are shown as horizontal lines with the associated *p* values.


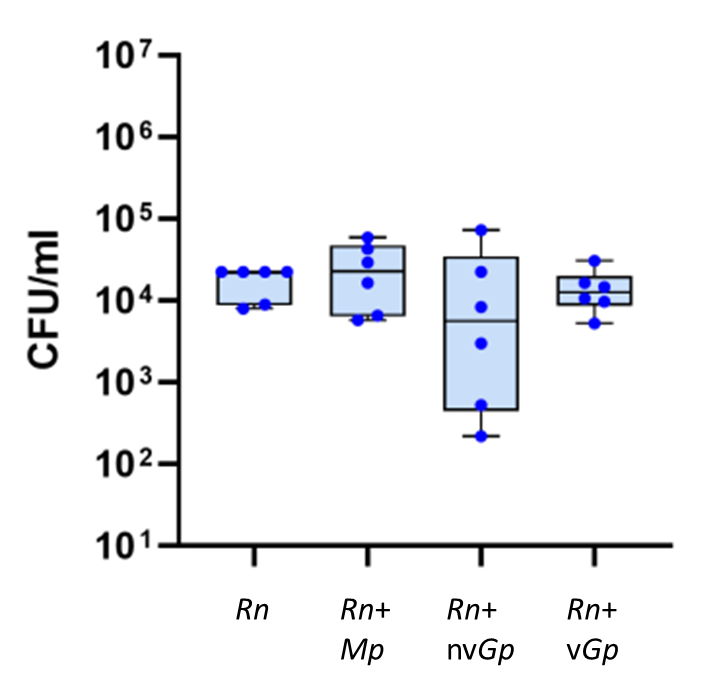


Figure S7. *Rothia nasimurium* adhesion after 2 h of incubation with non-differentiated porcine nasal organoids (PNOs) individually or in co-culture with selected strains. Non-differentiated PNOs were inoculated with 10^4^ CFU/ml of *R. nasimurium* (*Rn*) alone or in combination with approximately 10^3^ CFU/ml of *Moraxella pluranimalium* (*Mp*), 10^3^ CFU/ml of non-virulent *Glaesserella parasuis* (nv*Gp*) or with 10^5^ CFU/ml of virulent *G. parasuis* (v*Gp*). After 2 h of incubation, attached *Rn* was quantified by dilutions and plating after washing to eliminate unbound bacteria. Three independent experiments were performed with duplicate wells and each dot in the plot represents individual well results.


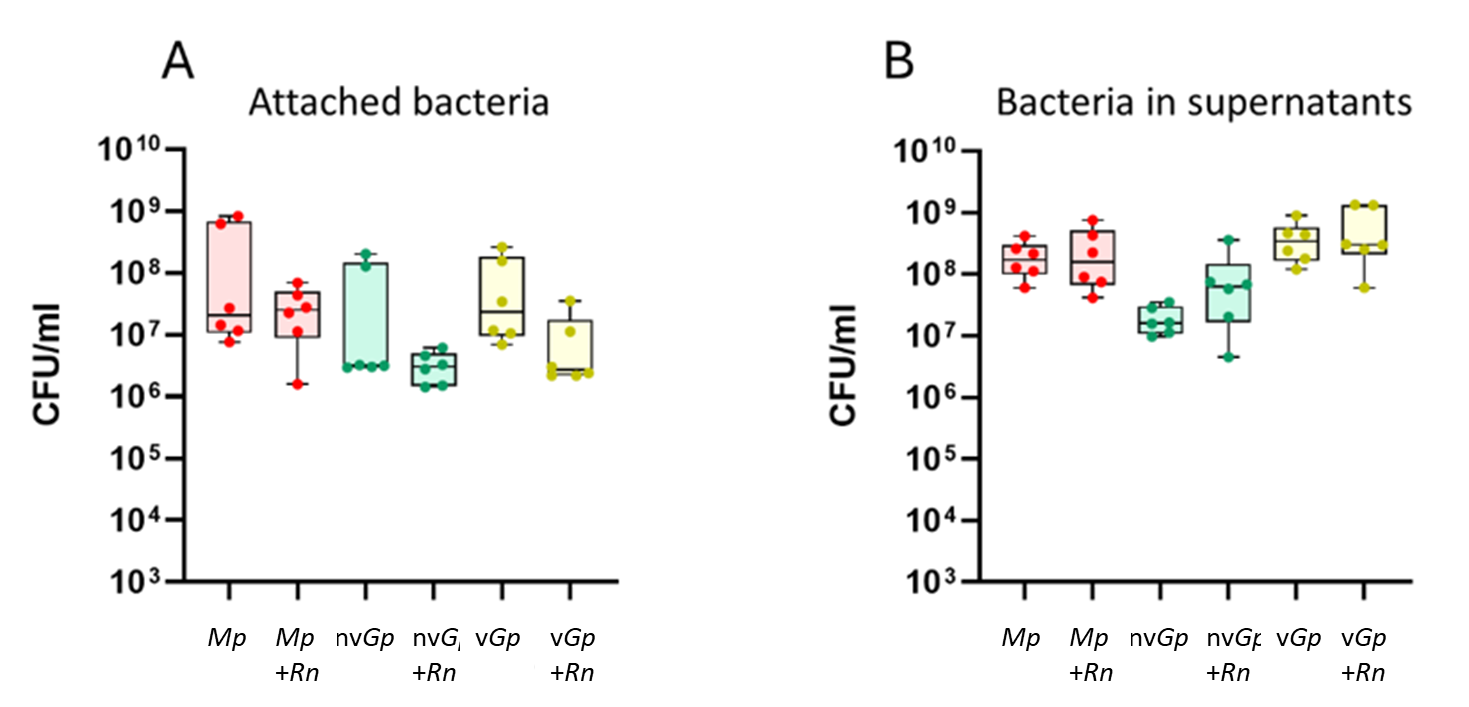


**Figure S8. Co-culture with Rothia nasimurium in the non-differentiated Porcine Nasal Organoids (PNOs) after overnight incubation.** Non-differentiated PNOs were incubated overnight with approximately 10^3^ CFU/ml of *Moraxella pluranimalium* (*Mp*), 10^3^ CFU/ml of non-virulent *Glaesserella parasuis* (nv*Gp*) or with 10^5^ CFU/ml of virulent *G. parasuis* (v*Gp*) individually or in combination with 10^4^ CFU/ml of *R. nasimurium* (*Rn*). a) As a measure of colonization capacity, attached *Mp* (in red), n*vG* (in green) and v*Gp* (in yellow) were quantified after individual incubation or co-cultured with *Rn*. b) Quantification of *Mp* (red), nv*Gp* (green) and v*Gp* (yellow) in the supernatant. Each dot represents a replicate from three different experiments of PNOs coming from two animals. Two replicates were included in each experiment.


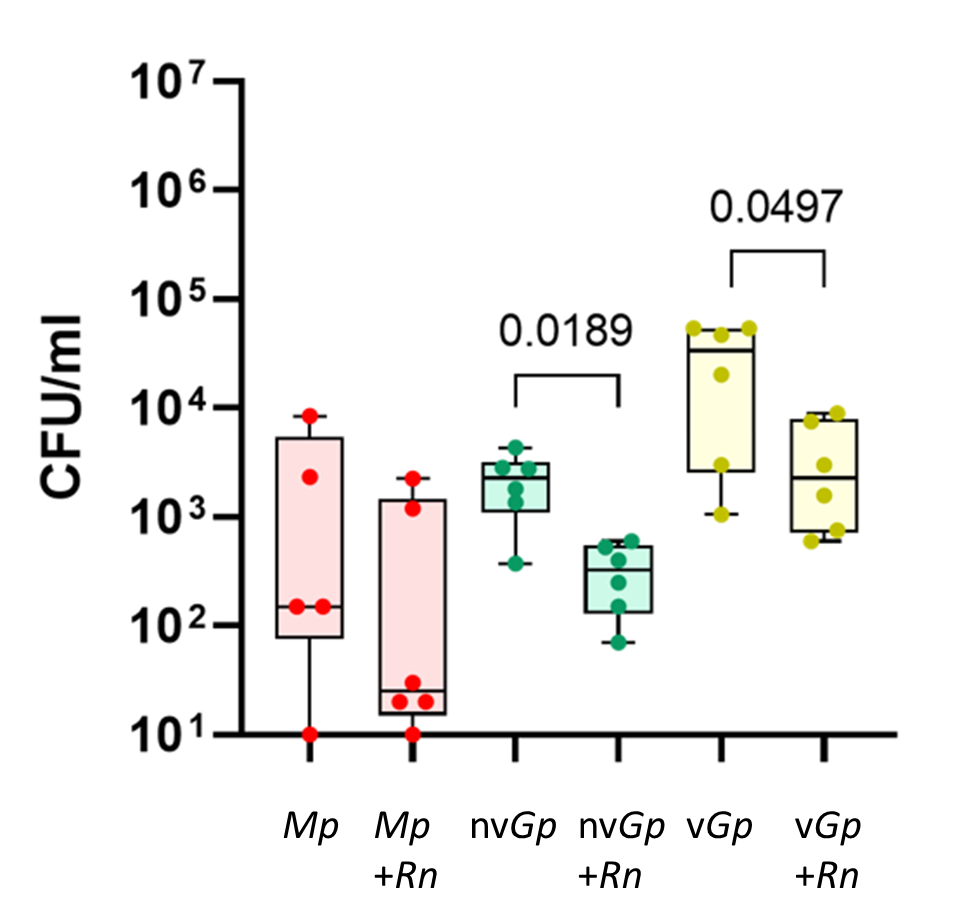


**Figure S9.** Adhesion of *Moraxella pluranimalium* and non-virulent and virulent strains of *Glaesserella parasuis* co-cultured with *Rothia nasimurium* in non-differentiated Porcine Nasal Organoids (PNOs). Non-differentiated PNOs were incubated for 2h with approximately 10^3^ CFU/ml of *Moraxella pluranimalium* (*Mp*), 10^3^ CFU/ml of non-virulent *Glaesserella parasuis* (nv*Gp*) or with 10^5^ CFU/ml of virulent *G. parasuis* (v*Gp*) alone or in combination with 10^5^ CFU/ml of *R. nasimurium* (*Rn*). Adherent bacteria were quantified (CFU/ml) after eliminating non-attached ones by washing. Quantification of adherent *Mp* (in red), nv*Gp* (in green) and v*Gp* (in yellow) after individual incubation or co-cultured with Rn are shown. Each dot represents a replicate from three different experiments of PNOs coming from two animals. Two replicates were included in each experiment. Significant differences are shown in the graph as *p* values using Welch’s t test.


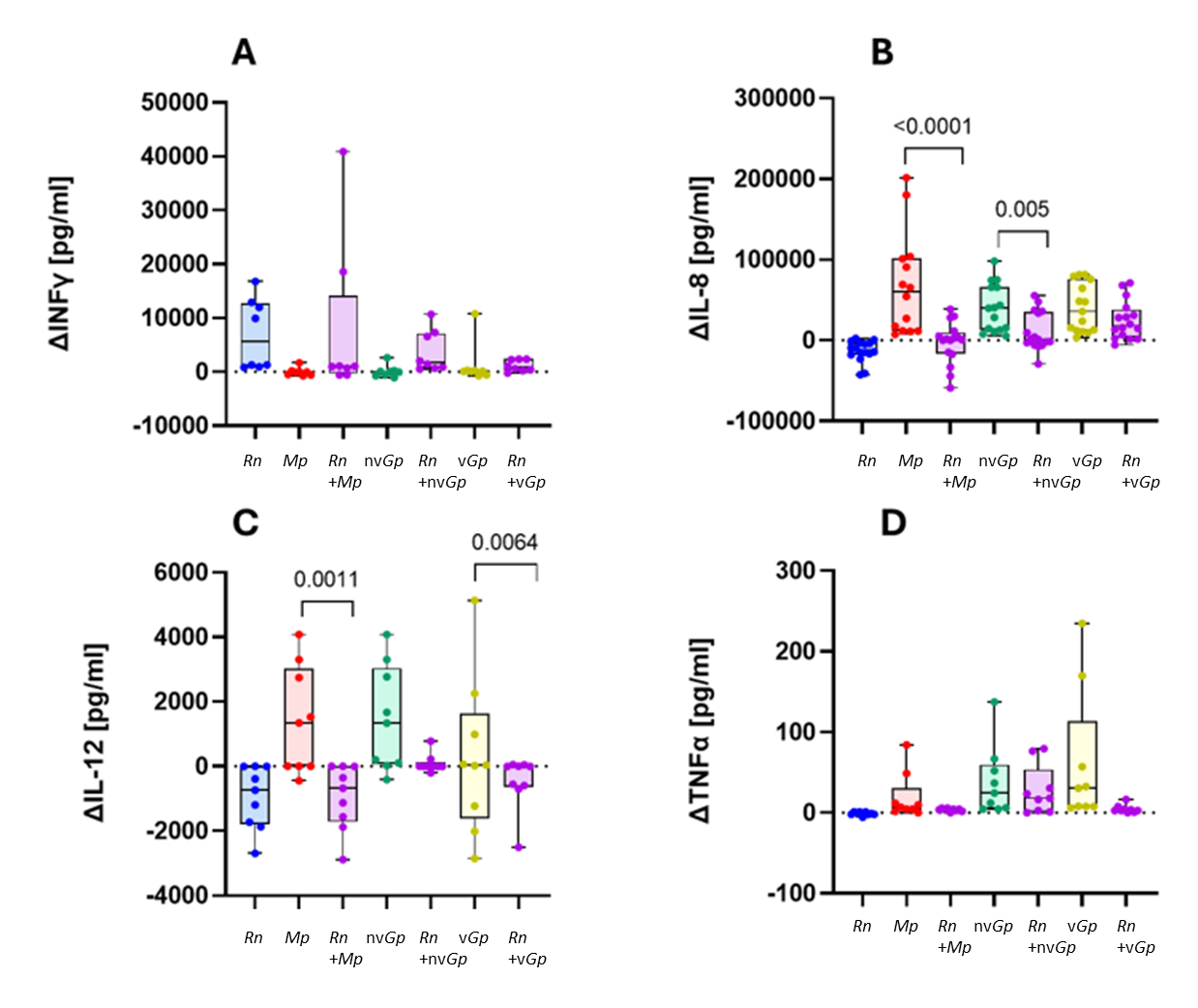


Figure S10. Interleukins and cytokines concentrations secreted by the non-differentiated Porcine Nasal Organoids (PNOs) when stimulated with the bacteria. Non-differentiated PNOs were incubated with 10^4^ CFU/ml of *R. nasimurium* (*Rn*), 10^3^ CFU/ml of *Moraxella pluranimalium* (*Mp*), 10^3^ CFU/ml of non-virulent *Glaesserella parasuis* (nv*Gp*) or with 105 CFU/ml of virulent *G. parasuis* (v*Gp*) individually or in combinations. After overnight incubation supernatants were recovered for cytokine quantification. Results are shown as the delta of each interleukin and cytokine, subtracting the negative control (PNOs without inoculation, basal levels of the secretion) in pg/ml. a) Secretion of interleukin 8 (IL-8), b) Secretion of interleukin 12 (IL-12), c) Secretion of Tumor Necrosis Factor alpha (TNFα) and d) Secretion of Interferon gamma (INFγ). Each dot represents a replicate from three different experiments of PNOs coming from two animals. Two replicates were included in each experiment. Kruskal-Wallis multiple comparison with Benjamini, Krieger and Yekutieli post-hoc test was used to compare the bacterial concentrations. Significant difference are shown as horizontal lines with the associated *p* values.


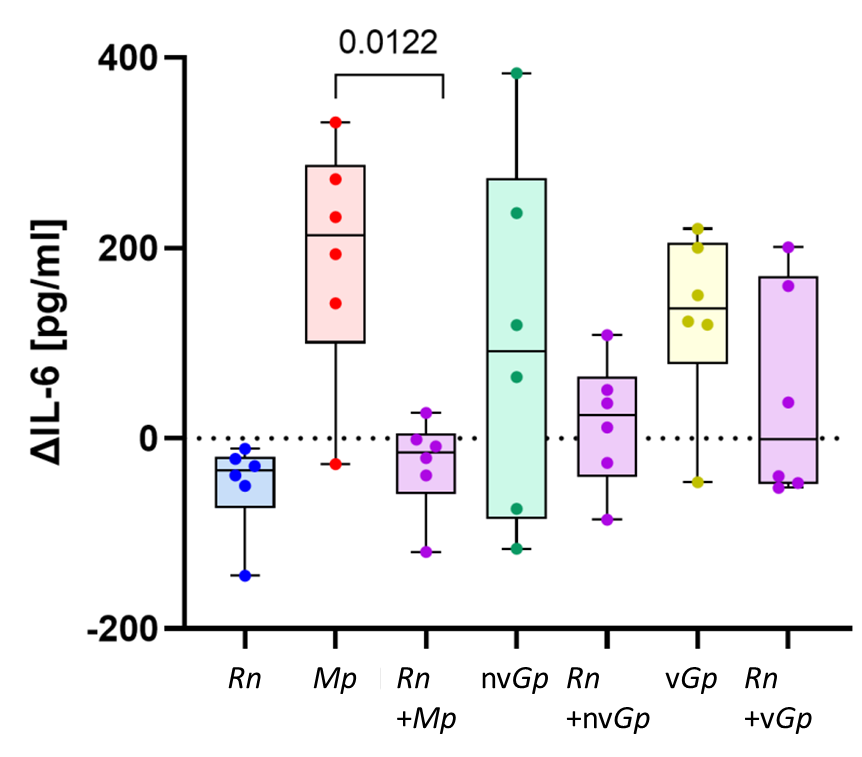


Figure S11. Interleukin 6 (IL-6) concentrations by the non-differentiated Porcine Nasal Organoids (PNOs) when stimulated with the bacteria. Non-differentiated PNOs were incubated with 10^4^ CFU/ml of *Rothia nasimurium* (*Rn*), 10^3^ CFU/ml of *Moraxella pluranimalium* (*Mp*), 10^3^ CFU/ml of non-virulent *Glaesserella parasuis* (nv*Gp*) or with 10^5^ CFU/ml of virulent *G. parasuis* (v*Gp*) individually or in combinations. After overnight incubation supernatants were recovered for cytokine quantification. Results are shown as the delta of interleukin 6 (IL-6), subtracting the negative control (PNOs without inoculation, basal levels of the secretion) in pg/ml. Each dot represents a replicate from three different experiments of PNOs coming from two animals. Two replicates were included in each experiment. Kruskal-Wallis multiple comparison with Benjamini, Krieger and Yekutieli post-hoc test was used to compare the bacterial concentrations. Significant difference is shown as a horizontal line with the associated *p* value.

Movie S1.

Cilia oscillations in 3D porcine nasal organoids (PNOs).
